# Comparison of radiological and clinical outcomes of cervical laminoplasty versus lateral mass screw fixation in patients with ossification of the posterior longitudinal ligament

**DOI:** 10.1186/s12891-024-07385-2

**Published:** 2024-04-26

**Authors:** Tao Liu, Jianzhou Zhang, Longlian Deng, Mengzi He, Shuo Tian, Wenyuan Ding, Zheng Wang, Dalong Yang

**Affiliations:** 1 Department of Spinal Surgery, The Third Hospital of Hebei Medical University, 139 Ziqiang Road, Shijiazhuang, 050051 PR China; 2 Department of Orthopedics, Xuanwu Hospital, Capital Medical University, No.45 Changchun Street, Xicheng District, Beijing, 100053 China; 3 Department of gastrointestinal Surgery, Bayannur hospital, Inner Mongolia Medical University, No. 98 Ulanbuhe Street, Linhe District, Bayannur, 015000 China

**Keywords:** Posterior longitudinal ligament ossification, Laminoplasty, Lateral mass screw fixation, Sagittal alignment, Clinical outcome

## Abstract

**Purpose:**

This study aimed to compare cervical sagittal parameters and clinical outcomes between patients undergoing cervical laminoplasty(CL) and those undergoing lateral mass screw fixation(LMS).

**Methods:**

We retrospectively studied 67 patients with multilevel ossification of the posterior longitudinal ligament (OPLL) of the cervical spine who underwent lateral mass screw fixation (LMS = 36) and cervical laminoplasty (CL = 31). We analyzed cervical sagittal parameters (C2-7 sagittal vertical axis (C2-7 SVA), C0-2 Cobb angle, C2-7 Cobb angle, C7 slope (C7s), T1 slope (T1s), and spino-cranial angle (SCA)) and clinical outcomes (visual analog scale [VAS], neck disability index [NDI], Japanese Orthopaedic Association [JOA] scores, recovery rate (RR), and minimum clinically significant difference [MCID]). The cervical sagittal parameters at the last follow-up were analyzed by binary logistic regression. Finally, we analyzed the correlation between the cervical sagittal parameters and each clinical outcome at the last follow-up after surgery in both groups.

**Results:**

At the follow-up after posterior decompression in both groups, the mean values of C2-C7 SVA, C7s, and T1s in the LMS group were more significant than those in the CL group (*P* ≤ 0.05). Compared with the preoperative period, C2-C7 SVA, T1s, and SCA gradually increased, and the C2-C7 Cobb angle gradually decreased after surgery (*P* < 0.05). The improvement in the JOA score and the recovery rate was similar between the two groups, while the improvement in the VAS-N score and NDI score was more significant in the CL group (*P* = 0.001; *P* = 0.043). More patients reached MCID in the CL group than in the LMS group (*P* = 0.036). Binary logistic regression analysis showed that SCA was independently associated with whether patients reached MCID at NDI postoperatively. SCA was positively correlated with cervical NDI and negatively correlated with cervical JOA score at postoperative follow-up in both groups (*P* < 0.05); C2-7 Cobb angle was negatively correlated with cervical JOA score at postoperative follow-up (*P* < 0.05).

**Conclusion:**

CL may be superior to LMS in treating cervical spondylotic myelopathy caused by OPLL. In addition, smaller cervical SCA after posterior decompression may suggest better postoperative outcomes.

## Introduction

Ossification of the posterior longitudinal ligament (OPLL) is a multifactorial disease caused by ectopic osteophytes and calcification of the posterior longitudinal ligament [1], which results in the formation of a bony mass that causes varying degrees of spinal cord compression and deterioration of neurological function [2]. Asymptomatic patients can be temporarily treated conservatively, but patients with symptomatic OPLL require surgical treatment. Posterior decompression surgery has been generally recognized as a commonly used surgical procedure, including laminoplasty, laminectomy, and laminectomy and fusion(LF), which can adequately decompress the spinal cord. However, it cannot directly remove the OPLL [3–5]. In patients with cervical spondylotic myelopathy due to multisegmental OPLL, posterior decompression surgery may be more reliable and effective compared to anterior decompression surgery because it has a lower risk of complications and can avoid the need for anterior surgery or significant problems associated with stent-grafts, such as graft dislocation, pseudarthrosis, or cerebrospinal fluid leakage [6, 7]. There are two main types of posterior cervical decompression procedures commonly performed in clinical practice today: laminoplasty and laminectomy combined with internal fixation and fusion. The clinical and radiological results regarding the two posterior decompression procedures have received extensive attention.

CL has been widely reported to reduce postoperative anterior cervical lordosis and postoperative lordosis deformities that would result in neck pain and cervical disability [8–10]. In 2003, Huang et al. [11]first reported that laminectomy with fusion (LF) had exemplary clinical and radiological outcomes in multisegmental spinal cervical spondylosis in neutral or anteriorly convex sagittal position. Huang [11] and Liu [12] et al. also reported that cervical laminectomy combined with internal fixation could improve and maintain alignment of anterior cervical lordosis and that posterior cervical internal fixation could provide greater biomechanical strength, could effectively prevent the progression of ossification of the posterior longitudinal ligaments of the cervical spine, and reduce lordosis and instability after laminectomy. Recently, Ha et al. [13]found that laminoplasty was superior to laminectomy combined with internal fixation fusion in terms of preserving cervical ROM, preoperative cervical pronation, and minimizing cervical disability and that the increased thickness of OPLL was inhibited by the stability obtained through increased instrumented fixation fusion. More literature on the sagittal alignment and clinical outcomes of CL combined with LF in treating cervical spondylotic myelopathy due to multisegmental OPLL needs to be published. These controversies persist due to the need for studies comparing CL and LF performed by the same group of surgeons [14, 15].

In recent years, the cervical sagittal balance has been identified as an essential determinant of radiological and clinical outcomes after cervical and thoracolumbar spine surgery [16]. Therefore, we chose to compare cervical laminoplasty and lateral mass screw fixation for patients with cervical spondylotic myelopathy due to multisegmental OPLL. We aimed to compare the sagittal alignment and clinical outcomes of the cervical spine between patients undergoing CL and those undergoing LMS.

## Methods

### Patient demographics

This study was approved by the local ethics committee and institutional review board (W2021-041-1). This paper was a retrospective study. Sixty-seven patients with multisegmental cervical posterior longitudinal ligament ossification were included from January 2020 to January 2022. Patients who underwent surgery were divided into two groups: lateral mass screw fixation (LMS) (*n* = 36) and cervical laminoplasty (CL) (*n* = 31). Inclusion criteria: OPLL with multisegmental lesions diagnosed by CT or MRI; complete imaging and clinical data; patients with > 12-month postoperative follow-up. Exclusion criteria: history of cervical spine surgery; patients with ossification of the ligamentum flavum; spinal injury, tumor, infection, congenital disease, or inflammatory arthritis (including ankylosing spondylitis and rheumatoid arthritis); failure to show T1 vertebrae on cervical spine X-ray for various reasons (e.g., obese, short-necked patients), etc.

### Operative technique

All procedures included in this study were performed under the supervision of the same surgeon (YDL).

1. Cervical laminoplasty (CL): The patient is placed prone under general anesthesia, routinely disinfected, and sterile towels are laid. A straight incision is made in the posterior mid-cervical spine, and the skin, subcutaneous tissue, and collateral ligament are incised. Subperiosteal dissection revealed the spinous processes and laminae of the target cervical segment and reached the bilateral laminae, articular processes, and lateral mass. The less symptomatic side was selected as the portal side at the medial edge of the articular processes of the vertebral plates on both sides of the target segment. The outer cortical bone of the vertebral plates was ground away with a grinding drill to form a V-shaped bone groove. The more symptomatic side is the open side, and the inner and outer cortical layers of the vertebral plate are ground through, with an opening angle of about 45 to 60 degrees. After spinal canal decompression, the dural sac is exposed and cleared of connective tissue posteriorly to ensure good pulsation and complete decompression of the dural sac. A miniature titanium plate is inserted in the decompressed segment to keep the “door” open. The adhesions between the lamina and dura are loosened, the dural sac is covered with a gelatin sponge, and a drainage tube is placed. Finally, the incision is closed layer by layer. For 24 h after surgery, the patient is closely monitored for changes in limb movement and sensation. All patients were required to wear a Philadelphia neck brace for approximately 2–4 weeks and were advised to perform appropriate physical function exercises. Follow-up radiographs were performed at 1, 3, 6, and 12 months postoperatively and every six months after that.
2. Lateral mass screw fixation (LMS): The patient is placed prone under general anesthesia, routinely disinfected, and sterile towels are laid. A straight posterior cervical incision is made, and the skin, subcutaneous tissue, and collateral ligament are incised. Subperiosteal dissection revealed the spinous processes and laminae of the target cervical segment and reached the bilateral laminae, articular processes, and lateral mass. Lateral mass screws are inserted in the lateral blocks of the vertebral plates on both sides of the target segment, and the connecting rods are attached bilaterally. The target stage spinous process and bilateral laminae were excised and thoroughly decompressed, and the dura was seen to be intact and well pulsed intraoperatively. The surgical field was flushed with physiological saline, and the internal fixation was well positioned on fluoroscopy. A drainage tube was placed, and the surgical opening was finally closed layer by layer. The patient needs to be closely monitored for 24 h postoperatively for changes in limb movement and sensation. All patients were required to wear a Philadelphia neck brace for approximately 2–4 weeks and were advised to perform appropriate physical function exercises. Follow-up radiographs were performed at 1, 3, 6, and 12 months postoperatively and every six months after that.

### Radiographic assessment

**Fig. 1:**
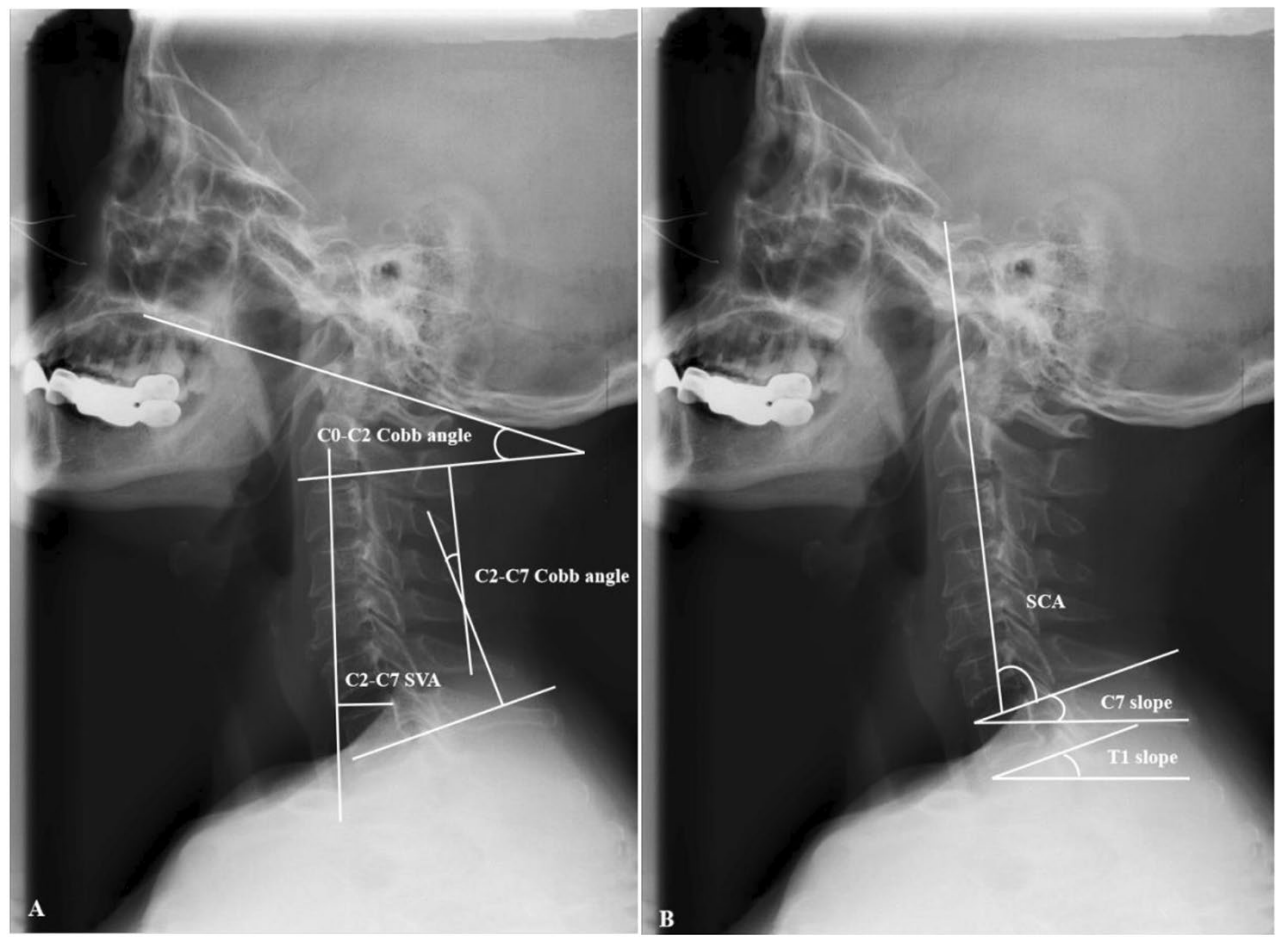
Schematic diagrams of the parameters. Radiographic measurements of cervical sagittal parameters in this study: *C0-C2 Cobb angle*: angle between the McGregor line and the C2 lower end plate, *C2–C7 Cobb angle*: angle between the lower plate of C2 and the lower plate of C7, *C2–7 sagittal vertical axis(C2–7 SVA)*: the distance from the posterior, superior corner of C7 to the plumbline from the centroid of C2, *C7 Slope*: the distance from the posterior, superior corner of C7 to the plumbline from the centroid of C2, *T1 Slope*: angle between a horizontal line and the superior endplate of T1, and *Spino-cranial angle(SCA)*: the angle is defined between the C7 slope and the straight line joining the middle of the C7 end plate and the middle of the sella turcica

Figure 1 shows lateral cervical spine films were obtained for all subjects using the Picture Archiving and Communication System (PACS), with the issues in a neutral position and looking straight ahead. Cervical spine radiological measurements were performed by assessing the following parameters: (1) C2-C7 sagittal vertical axis (C2-C7 SVA), (2) C0-C2 Cobb angle, (3) C2-C7 Cobb angle, (4) T1 slope(T1s), (5) C7 slope(C7s), and (6) spino-cranial angle (SCA). The measured variables are defined in Fig. 1 [13, 17].

### Clinical outcome assessment

Medical information and imaging manifestations such as patient age, gender, height, weight, BMI, history of hypertension, history of diabetes mellitus, type of OPLL, surgical segment, operation time, and bleeding amount were investigated. Clinical outcomes were assessed using the patient-reported visual analog scale for neck pain (VAS-N) (range 0–10) and arm pain (VAS-A) (range 0–10), neck disability index (NDI) (range 0–50), and Japanese Orthopaedic Association (JOA) score (score 0–17). These data were available for all patients preoperatively and at least 24 months postoperatively. Neurological outcomes were assessed according to the JOA score [18]. The postoperative recovery rate(RR) was calculated as follows: RR(%) = (postoperative JOA score - preoperative JOA score) / (17 - preoperative JOA score). The minimal clinically significant difference (MCID) was defined as the slightest change that the patient could identify as “clinically meaningful” to assess the success of the intervention. We reviewed previous studies [19, 20], which reported an MCID of 2 for defining VAS scores, 6 for NDI scores, 2.5 for JOA scores, and 52.8% for JOA recovery rates. In the present study, we used the MCID for each clinical outcome as a reference standard to measure whether the change in each clinical outcome between the two groups of posterior decompression surgery was significant enough to produce clinical differences.

### Statistical analysis

All values are expressed as mean ± standard deviation or percentage. Normally distributed data were compared using the independent samples t-test, χ^2^ test, or Fisher’s exact test. Non-normally distributed data were evaluated using nonparametric test analysis, and the Mann-Whitney U test was used to assess differences between the two groups. Normally distributed data within the two groups were compared, e.g., preoperative, 12-month postoperative, and final follow-up cervical sagittal parameters were compared using repeated measures analysis of variance (ANOVA). A paired-sample t-test was used for preoperative and postoperative clinical outcomes. The cervical sagittal parameters at the last follow-up were analyzed using binary logistic regression to evaluate whether the NDI score achieved MCID. The results were expressed as the ratio (OR) and 95% confidence interval (CI). Finally, Spearman correlation analysis assessed the relationship between cervical sagittal parameters and VAS, NDI, and JOA scores at the last follow-up. All statistical analyses were performed using SPSS software (version 26.0; SPSS et al., USA). A *p*-value < 0.05 was considered statistically significant.

## Results

### General data

Sixty-seven patients (51 males and 16 females) with cervical OPLL were included in this study. The patients were divided into two groups according to the surgical modality: 36 patients (25 males and 11 females) were treated surgically for LMS, and 31 patients (26 males and 5 females) were treated surgically for CL. There were more male patients with cervical OPLL than female patients. The characteristics of these patients were summarized (Table 1). There were no statistically significant differences between the two groups regarding age, gender, height, weight, BMI, history of diabetes mellitus, and history of hypertension (*P* > 0.05). The mean age at the time of surgery was 59.19 years (range 35–78 years).

**Table 1:** Patient demographics and clinical characteristics

	LMS	CL	*P*-Value
No. of patients	36	31	
Age(year) Male/Female Weight(kg) Height(cm) BMI(kg/m^2^) Hypertension(%) DM(%)	58.53 ± 9.55 25/11 71.58 ± 12.35 167.69 ± 9.36 25.34 ± 3.11 12(33.3) 6(16.7)	59.97 ± 8.07 26/5 75.18 ± 10.45 167.90 ± 5.69 26.57 ± 2.66 13(41.9) 2(6.5)	0.511 0.167 0.207 0.455 0.089 0.468 0.270
Type of OPLL			0.873
Continuous Segmental Mixed	7 16 13	5 13 13	
**Operation level**			
C2/3/4/5 C3/4/5/6 C4/5/6/7 C2/3/4/5/6 C3/4/5/6/7 C2/3/4/5/6/7	2 18 3 1 10 2	0 20 1 0 8 2	
**Mean**	**4.36**	**4.29**	**0.680**
complications (*N*)	0	0	0.764
Spinal cord injury C5 nerve palsy CSF leakage Screw looseness/failure Wound infection Postoperative Hematoma	4 0 0 1 0 140.89 ± 35.86	2 0 0 1 1 138.26 ± 39.07	
Operation time (min)	330.83 ± 132.22	323.55 ± 146.25	0.840
Bleeding amount (ml)			0.739

All data are expressed as mean ± SD unless otherwise noted

*OPLL* Ossification of posterior longitudinal ligament, *LMS* Lateral mass screw fixation, *CL* Cervical laminoplasty

*P < 0.05

Segmental and mixed OPLL were the two most common types in surgical patients. The average number of operated-upon levels was 4.36 in the LMS group and 4.29 in the CL group. OPLL in the upper cervical spine (C2) or cervicothoracic region (C7) was more common in the LMS group (17/ 36,47.22%) than in the CL group (11/31,35.48%) and the C3-C6/C7 region was the most common segmental region for surgical operations in both groups. However, there was no statistical difference between the number of surgically operated segments in the two groups (*P* > 0.05). There was no statistical difference in the operative time and the amount of bleeding between the two groups (*P* > 0.05). The demographic characteristics of the patients in this study are shown in Table 1.

### Radiological results

Table 2 summarizes the values of cervical sagittal parameters in the LMS and CL groups of the cervical spine. At the last follow-up, the mean C2-C7 SVA values were more significant in the LMS group than in the CL group (35.23 ± 8.40 vs. 31.15 ± 8.25, *P* = 0.05); the mean C7s and T1s values were more significant in the LMS group than in the CL group (27.76 ± 7.90 vs. 23.39 ± 6.64, *P* < 0.05; 31.43 ± 6.72 vs. 27.84 ± 5.13, *P* < 0.05). There was no statistical difference between the two groups for each cervical sagittal parameter preoperatively, 12 months postoperatively, and at the final follow-up (*P* > 0.05).

**Table 2:** Comparison of radiological outcome according to surgical technique

	LMS	CL	*P*-Value
C0–C2 Cobb angle (°)			
Pre Post 12 m F/U Pre VS. Post 12 m Pre VS. F/U	18.19 ± 7.54 19.05 ± 7.36 21.00 ± 7.53 0.002* <0.001*	19.72 ± 6.05 20.74 ± 7.36 21.77 ± 8.28 0.421 0.115	0.370 0.352 0.697
C2–C7 Cobb angle (°)			
Pre Post 12 m F/U Pre VS. Post 12 m Pre VS. F/U	14.03 ± 11.19 11.12 ± 10.33 9.86 ± 9.75 <0.001* <0.001*	13.76 ± 7.51 12.66 ± 7.13 9.07 ± 7.86 0.013* <0.001*	0.906 0.477 0.719
C2-C7 SVA (mm)			
Pre Post 12 m F/U Pre VS. Post 12 m Pre VS. F/U	25.14 ± 8.09 30.24 ± 7.84 35.23 ± 8.40 <0.001* <0.001*	23.72 ± 7.49 27.02 ± 7.41 31.15 ± 8.25 <0.001* <0.001*	0.461 0.089 0.050*
C7 slope(°)			
Pre Post 12 m F/U Pre VS. Post 12 m Pre VS. F/U	23.87 ± 7.41 25.40 ± 7.91 27.76 ± 7.90 <0.001* <0.001*	21.89 ± 5.17 22.89 ± 5.19 23.39 ± 6.64 <0.001* 0.228	0.205 0.126 0.017*
T1 slope(°)			
Pre Post 12 m F/U Pre VS. Post 12 m Pre VS. F/U	27.13 ± 6.86 28.89 ± 7.00 31.43 ± 6.72 <0.001* <0.001*	25.34 ± 4.30 26.84 ± 4.09 27.84 ± 5.13 <0.001* 0.014*	0.200 0.142 0.018*
SCA(°)			
Pre Post 12 m F/U Pre VS. Post 12 m Pre VS. F/U	76.69 ± 7.62 86.47 ± 7.68 94.92 ± 7.86 <0.001* <0.001*	78.24 ± 5.95 84.74 ± 6.07 92.74 ± 6.07 <0.001* <0.001*	0.392 0.307 0.214

All data are expressed as mean ± SD unless otherwise noted

*Pre* Preoperative, *Post* Postoperative, *F/U* Follow up, *C2–7 SVA* C2–7 sagittal vertical axis, *Postop* Postoperative, *SCA* Spino-cranial angle, *m* month, *LMS* Lateral mass screw fixation, *CL* Cervical laminoplasty

*P < 0.05

**Fig. 2:**
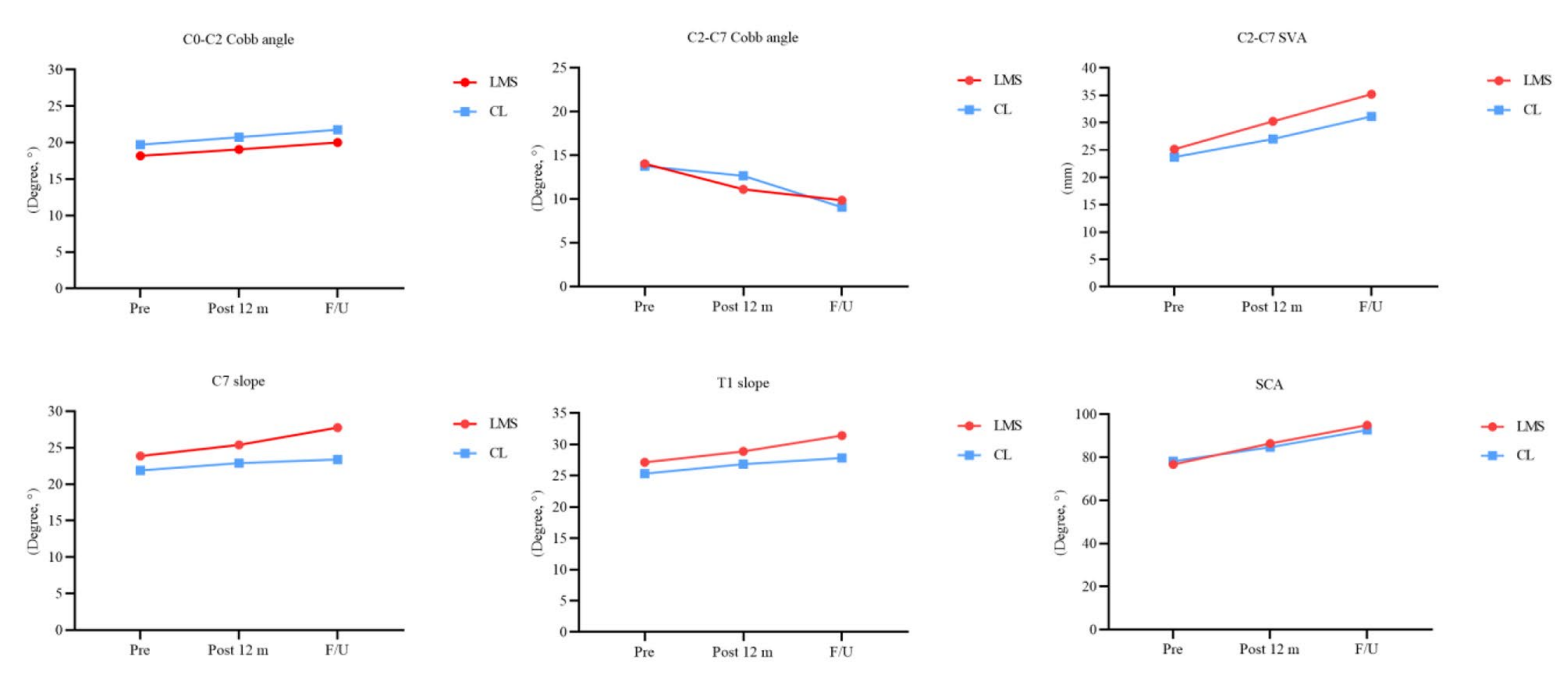
The change in the radiographic parameters. *C2–7 SVA* C2–7 sagittal vertical axis, *SCA* Spino-cranial angle

At follow-up after posterior decompression in both groups, the C2-C7 SVA, T1s, and SCA gradually increased, and the C2-C7 Cobb angle gradually decreased compared with the preoperative cervical radiological parameters. Table 3 summarizes the changes in each cervical sagittal parameter between the two groups preoperatively, at 12 months postoperatively, and at the final follow-up. In the LMS group, the C0-C2 Cobb angle was significantly greater postoperatively and at the last follow-up compared with preoperatively (18.19 ± 7.54 vs. 19.05 ± 7.36, *P* = 0.002; 18.19 ± 7.54 vs. 21.00 ± 7.53, *P* < 0.001). Postoperative and final follow-up C7s were significantly greater (23.87 ± 7.41 vs. 25.40 ± 7.91, *P* < 0.001; 23.87 ± 7.41 vs. 27.76 ± 7.90, *P* < 0.001). Postoperative and final follow-up T1s were significantly greater (27.13 ± 6.86 vs. 28.89 ± 7.00, *P* < 0.001; 27.13 ± 6.86 vs. 31.43 ± 6.72, *P* < 0.001). The SCA was also significantly greater postoperatively and at the last follow-up (76.69 ± 7.62 vs. 86.47 ± 7.68, *P* < 0.001; 76.69 ± 7.62 vs. 94.92 ± 7.86, *P* < 0.001). The C2-C7 Cobb angle was significantly decreased postoperatively and at the last follow-up compared to preoperatively (14.03 ± 11.19 vs. 11.12 ± 10.33, *P* < 0.001; 14.03 ± 11.19 vs. 9.86 ± 9.75, *P* < 0.001). In the CL group, there was no statistically significant difference in postoperative and final follow-up C0-C2 Cobb angles compared to preoperative (19.72 ± 6.05 vs. 20.74 ± 7.36, *P* = 0.421; 19.72 ± 6.05 vs. 21.77 ± 8.28, *P* = 0.115). Compared with preoperative, postoperative C7s were significantly greater (21.89 ± 5.17 vs. 22.89 ± 5.19, *P* < 0.001), while there was no statistically significant difference in the last follow-up C7s (21.89 ± 5.17 vs. 23.39 ± 6.64, *P* = 0.228). Postoperative and last follow-up T1s were significantly greater (25.34 ± 4.30 vs. 26.84 ± 4.09, *P* < 0.001; 25.34 ± 4.30 vs. 27.84 ± 5.13, *P* = 0.014). The SCA was also significantly greater postoperatively and at the last follow-up (78.24 ± 5.95 vs. 84.74 ± 6.07, *P* < 0.001; 78.24 vs. 92.74 ± 6.07, *P* < 0.001). Compared with the preoperative period, the C2-C7 Cobb angle was significantly decreased postoperatively and at the last follow-up (13.76 ± 7.51 vs. 12.66 ± 7.13, *P* = 0.013; 13.76 ± 7.51 vs. 9.07 ± 7.86, *P* < 0.001). During the postoperative follow-up, patients in the LMS group had a forward cervical alignment compared to those in the CL group. The C2-7 SVA in the LMS group increased from 25.14 ± 8.09 mm preoperatively to 30.24 ± 7.84 mm at 12 months postoperatively and 35.23 ± 8.40 mm at the last follow-up. On the other hand, the C2-7 SVA in the CL group increased from 23.72 ± 7.49 mm to 27.02 ± 7.41 mm at 12 months postoperatively and to 31.15 ± 8.25 mm at the last follow-up (Table 2). The changes in the above cervical sagittal parameters are shown as a line graph (Fig. 2).

**Table 3:** Comparison of clinical outcome according to surgical technique

	LMS	CL	*P*-Value
VAS score, neck			
Pre F/U Pre VS. F/U	4.28 ± 1.39 2.42 ± 1.05 <0.001*	3.61 ± 1.80 1.45 ± 1.18 <0.001*	0.062 0.001*
VAS score, arm			
Pre F/U Pre VS. F/U	5.42 ± 1.38 3.19 ± 1.01 <0.001*	5.38 ± 1.58 2.81 ± 1.25 <0.001*	0.738 0.175
NDI score			
Pre F/U Pre VS. F/U	25.06 ± 5.91 14.44 ± 6.37 <0.001*	23.06 ± 4.71 11.29 ± 3.65 <0.001*	0.175 0.043*
JOA score			
Pre F/U Pre VS. F/U	11.03 ± 1.63 14.03 ± 1.25 <0.001*	11.65 ± 1.52 14.55 ± 1.09 <0.001*	0.096 0.069
RR (%)	46.19 ± 30.59	50.58 ± 27.20	0.491

*VAS* Visual analog scale, *NDI* Neck disability index, *JOA* Japanese Orthopaedic Association score

*RR*, Recovery rate; *Pre*, Preoperative; *F/U*, Follow up, *LMS* Lateral mass screw fixation, *CL* Cervical laminoplasty

*P < 0.05

### Clinical outcome assessment

LMS and CL for OPLL significantly improved postoperative clinical outcomes (Table 3). At preoperative and final follow-up, the VAS-N, VAS-A, and NDI scores gradually decreased, and the JOA score gradually increased in both groups (*P* < 0.001). When comparing between groups, the VAS-N score (LMS 2.42 ± 1.05 vs. CL 1.45 ± 1.18) and the NDI score (LMS 14.44 ± 6.37 vs. CL 11.29 ± 3.65) were significantly higher in the LMS group than in the CL group at postoperative follow-up ( *P* = 0.001; *P* = 0.043). There were no significant statistical differences in mean VAS-A and JOA scores from preoperative to final follow-up in the LMS group compared with the CL group (*P* > 0.05). JOA recovery rates improved in the LMS and CL groups, but no significant differences were seen (*P* > 0.05). At postoperative follow-up after posterior decompression in both groups, 87.1% of patients in the CL group and 63.9% in the LMS group achieved MCID with NDI score, which was statistically different between the two groups (*P* = 0.03). No statistically significant differences were seen in the MCID of each clinical outcome score for the remaining postoperative follow-up between the two groups (*P* > 0.05) (Table 4).

**Table 4:** Comparison of MCID between patients followed up after cervical laminoplasty and lateral mass screw fixation

	LMS	CL	*P*-Value
VAS score, neck			
Achieved MCID(≥ 2)	23(63.9%)	24(77.4%)	0.228
VAS score, arm			
Achieved MCID(≥ 2)	25(69.4%)	27(87.1%)	0.084
NDI score			
Achieved MCID(≥ 6)	23(63.9%)	27(87.1%)	0.030*
JOA score			
Achieved MCID(≥ 2.5)	21(58.3%)	17(54.8%)	0.773
RR (%)			
Achieved MCID(≥ 52.8%)	14(38.9%)	15(48.4%)	0.434

*VAS* Visual analog scale, *NDI* Neck disability index, *JOA* Japanese Orthopaedic Association score

*RR*, Recovery rate; *LMS* Lateral mass screw fixation, *CL* Cervical laminoplasty; *MCID*, Minimum clinically important difference

*P < 0.05

We used MCID, which achieved an NDI score, as the outcome variable and included cervical sagittal parameters at the last follow-up for binary logistic regression analysis. Binary logistic regression analysis showed that SCA was independently associated with MCID regarding whether patients achieved NDI after surgery (OR = 0.905, *P* = 0.048)(Table 5).

**Table 5:** Binary logistic regression analysis of cervical sagittal parameters at the last follow-up

Parameters	OR	95%CI	*p*-Value
C0-C2 Cobb angle C2-C7 Cobb angle C2-C7 SVA C7 slope T1 slope SCA	1.092 1.022 0.992 0.892 1.067 0.905	0.991–1.203 0.945–1.105 0.898–1.095 0.749–1.061 0.884–1.288 0.819–0.999	0.076 0.590 0.866 0.196 0.500 0.048*

*C2–7 SVA* C2–7 sagittal vertical axis, *SCA* Spino-cranial angle, *m* month, *LMS* Lateral mass screw fixation, *CL* Cervical laminoplasty, *OR* Odds ratio, *CI* Confidence interval

*P < 0.05

In the LMS group, the C2-7 Cobb angle at the last follow-up was positively correlated with JOA (R^2^ = 0.629; *P* < 0.05). C2-7 SVA and SCA at the last follow-up were positively correlated with NDI (R^2^ = 0.392, *P* < 0.05; R^2^ = 0.345; *P* < 0.05) as well as SCA was negatively correlated with JOA (R^2^ = 0.672; *P* < 0.05) (Table 6). In the CL group, the C2-7 Cobb angle at the last follow-up was positively correlated with JOA (R^2^ = 0.462; *P* < 0.05). C7s at the final follow-up was negatively correlated with NDI (R^2^ = 0.407; *P* < 0.05). SCA was positively correlated with NDI (R^2^ = 0.527; *P* < 0.05) and negatively correlated with JOA (R^2^ = 0.642; *P* < 0.05) at the final follow-up (Table 7).

**Table 6:** Results of Spearman correlations between parameters of cervical alignment and clinical outcome at the final postoperative follow-up of lateral mass screw fixation

	C0-C2 Cobb angle	C2–C7 Cobb angle	C2-C7 SVA	C7 slope	T1 slope	SCA
VAS score, neck	-0.052	-0.045	0.284	0.057	0.046	0.060
VAS score, arm	0.126	-0.213	0.315	0.097	0.124	0.165
NDI score	0.094	-0.239	0.392*	-0.016	-0.146	0.345*
JOA score	−0.044	0.629*	−0.288	0.167	0.313	−0.672*

*VAS* Visual analog scale, *NDI* Neck disability index, *JOA* Japanese Orthopaedic Association score

*C2–7 SVA* C2–7 sagittal vertical axis, *SCA* Spino-cranial angle

*P < 0.05

**Table 7:** Results of Spearman correlations between parameters of cervical alignment and clinical outcome at the final postoperative follow-up of cervical laminoplasty surgery

	C0-C2 Cobb angle	C2–C7 Cobb angle	C2-C7 SVA	C7 slope	T1 slope	SCA
VAS score, neck	0.146	-0.065	0.206	-0.123	-0.127	0.253
VAS score, arm	-0.105	-0.135	0.012	-0.234	-0.154	0.153
NDI score	-0.076	-0.334	0.069	-0.407*	-0.348	0.527*
JOA score	0.149	0.462*	−0.100	0.264	0.220	−0.642*

*VAS* Visual analog scale, *NDI* Neck disability index, *JOA* Japanese Orthopaedic Association score

*C2–7 SVA* C2–7 sagittal vertical axis, *SCA* Spino-cranial angle

*P < 0.05

### Complications

Nine individuals (13.43%; *P* > 0.05) in this study had surgery-related complications. 4 (11.11%, 4/36) in the LMS group and 2 (6.45%, 2/31) in the CL group developed C5 nerve root palsy. They were instructed to strengthen muscle rehabilitation exercises and give methylcobalamin and vitamin B12 to nourish the nerve, and the patient’s discomfort disappeared at the follow-up after about three months. In addition, one patient in the CL group developed a postoperative hematoma. We immediately performed surgery to open the wound and remove the hematoma, and the patient recovered well at the postoperative follow-up. Surface wound infections occurred in 5 patients, and the wounds healed well after active antibiotics and dressing changes were given. (Table 1).

## Discussion

Cervical laminoplasty and cervical lateral mass screw fixation have been widely used in treating OPLL. In this study, we compared the sagittal alignment and clinical outcomes of the cervical spine in patients using both posterior cervical decompression procedures through clinical data and radiological examinations. Several studies have reported that CL may lead to posterior kyphosis and cause or exacerbate postoperative axial neck pain [8, 9, 17]. Kim et al. [21]also reported changes in cervical lordosis after laminoplasty with preoperative radiological parameters. The greater the preoperative extension capacity, the less the cervical lordosis reduction; the more significant the T1 slope, the greater the cervical lordosis reduction.

Moreover, studies have reported the treatment of OPLL by other posterior techniques, such as laminectomy and fusion (LF). Liu and Lee et al. [12, 22] said that cervical LF prevented the loss of cervical lordosis postoperatively and reduced cervical axial pain and disability. We compared clinical outcomes, including JOA, VAS and NDI, MCID, and radiological findings after multisegmental OPLL cervical laminoplasty with cervical lateral block screw internal fixation. This study illustrates that both surgical approaches significantly improved the clinical outcomes of patients postoperatively and the occurrence of significant changes in cervical sagittal parameters.

As we all know, increased C2-7 SVA, loss of C2-7 Cobb angle, and increased SCA are considered poor indicators of clinical prognosis after surgery [23–27]. Lin and Tang et al. [23, 28]reported that cervical laminoplasty can lead to malalignment and loss of cervical lordosis. Some literature reported [8, 23, 28]that loss of cervical lordosis or deformity of kyphosis of the cervical spine after CL surgery ranged from 33 to 70.7% of cases. In long-term follow-up studies, several researchers [7, 13]found that postoperative kyphosis and kyphotic changes were associated with poorer clinical outcomes. Several related studies have indicated that progressive kyphosis worsens neurological function in the late stages [9, 23]. Tang et al. reported that elevated C2-7 SVA after CL surgery negatively affects HRQOL scores and that disability in patients with positive sagittal malalignment severity is increased [23]. Wang et al., regarding SCA in cervical laminoplasty, found that SCA may be a good predictor for assessing sagittal balance and planning surgery and that SCA is in an appropriate fluctuating range to help maintain cervical spine stability [25, 27]. Other studies have reported that poor alignment or kyphotic deformity after cervical spine surgery is not associated with poor clinical outcomes [10, 12]. CL is generally not recommended for patients with significant preoperative kyphosis or at high risk of developing kyphosis due to postoperative kyphotic deformity, which aggravates neurological deficits [29]. Sakai et al. [7]also reported elevated C2-7 SVA after CL, which is unsuitable for patients with cervical spondylotic myelopathy with preoperative cervical sagittal imbalance. To avoid adverse clinical results caused by kyphosis after CL surgery, some researchers have proposed that posterior cervical laminectomy and internal fixation can reduce kyphosis and instability after laminectomy [12]. As a result, some spinal surgeons are increasingly opting for posterior cervical laminectomy and internal fixation, which they believe can prevent postoperative kyphosis, axial pain, and instability. In this study, at the last follow-up, we found a reduction in cervical lordosis in both groups but no significant difference between the two groups. After posterior decompression in both groups, we found that the C2-7 SVA was more significant in patients in the LMS group than in the CL group, i.e., the anterior cervical tilt was more pronounced in the LMS group. Based on these results, posterior decompression combined with lateral mass screw fixation may not maintain preoperative cervical alignment well, similar to Ha et al.‘s findings [13].

We analyzed the correlation between each cervical sagittal parameter and clinical outcomes at the final follow-up. We found poor sagittal alignment of the cervical spine, such as elevated SCA, elevated C2-7 SVA, and reduced C2-C7 Cobb angle, was associated with postoperative axial neck pain and neck dysfunction [13, 26, 27, 30]. In previous studies, LF significantly improved neck pain [11], while CL was associated with poor clinical outcomes such as shoulder and neck pain [11, 12]. However, our study found more significant improvement than LMS regarding VAS-N and cervical NDI after CL surgery. The two groups had no significant difference regarding postoperative cervical JOA and improvement in JOA RR%. Both posterior decompression procedures are accessed from the posterior aspect of the cervical spine, which inevitably causes unavoidable damage and disruption to the posterior muscle-ligament complex. Once the integrity of the cervical muscles is lost, cranial stability is diminished, inevitably leading to anterior axis displacement [31], which disrupts the original cervical alignment. The posterior cervical muscles are less exposed in CL than in LMS surgery. Because when performing LMS, we extensively dissect the posterior cervical muscles to determine the lateral mass screw entry point. As the degree of surgical invasion of the posterior muscle-ligament complex increases, the balance of the cervical sagittal plane decreases [26]. Despite the use of lateral mass screws to enhance the stability of the skeletal and joint structures, compensatory mechanisms may not be functional after damage to the muscle-ligament complex [13]. In our study, the increase in the C7s and T1s tended to be more significant in the LMS group than in the CL group to maintain the cervical lordosis curve. However this was insufficient to compensate for the poor alignment. Thus, damage to the posterior muscle-ligament complex and localized regional misalignments, such as reduced cervical lordosis and an excessive anterior tilt of the cervical spine, increase the energy expenditure related to maintaining horizontal gaze and head position [13]. These factors may explain why some patients experience axial neck pain and cervical dysfunction after posterior decompression surgery, particularly those who undergo LMS techniques. Therefore, sagittal imbalance after posterior cervical decompression may be associated with clinical outcomes, particularly postoperative axial neck pain, and cervical spine dysfunction.

Some recent studies have reported the relationship between cervical sagittal alignment and HRQOL [13, 23]. In our study, two posterior cervical decompression procedures were associated with improved VAS-N, VAS-A, NDI, and JOA scores. Several studies have reported that CL exacerbates or produces new axial neck pain postoperatively [32, 33]. We compared the VAS-N, VAS-A, NDI, and JOA scores and their MCID in patients with OPLL treated with laminoplasty versus lateral mass screw fixation. At the last follow-up, the VAS-N and NDI scores were significantly lower in the CL group than in the LMS group. Still, the VAS-A and JOA scores from preoperative to the last follow-up between the two groups’ improvements were not statistically different. Because of the statistical differences between the CL and LMS groups in terms of VAS-N scores and NDI scores, our results do not indicate a clinical advantage of the cervical laminoplasty technique over the cervical lateral mass screw technique in patients with OPLL who underwent both types of posterior decompression. Therefore, we included MCID for further comparative assessment of clinical differences between the two groups of procedures, and we found a high percentage of patients with MCID who achieved NDI scores after surgery in the CL group. Compared with the LMS group, our results showed that patients in the CL group showed significant improvements in VAS-N and NDI scores and a high percentage of MCID patients who achieved NDI. Therefore, this study may prefer CL to treat cervical spondylotic myelopathy caused by multisegmental cervical OPLL.

C5 nerve root palsy is a common complication after posterior cervical decompression, and it can directly affect patients’ postoperative outcomes and satisfaction [34]. A study conducted a meta-analysis on the prevalence of postoperative C5 nerve root palsy and demonstrated that LF was associated with a high prevalence of C5 nerve root palsy (11.0%) [35]. Our findings are consistent with previous studies [13, 35] that reported a lower prevalence of C5 nerve root palsy after CL (6.45%) than after LMS (11.11%).

This study has several limitations. First, the data were limited to the cervical spine. Pain, disability, and poor health were excluded due to the whole spine’s sagittal alignment and balance. Ideally, the whole-spine sagittal alignment would be assessed by using whole-spine standing X-rays. Second, a single-center study with a small sample size and a short follow-up was performed. Progression of kyphosis changes or OPLL after both posterior decompression procedures can affect clinical outcomes, so long-term follow-up of patients with OPLL is necessary.

## Conclusion

LMS and CL are two effective and successful posterior cervical decompression procedures for treating cervical spondylotic myelopathy due to OPLL. In addition, smaller cervical SCA after posterior decompression may suggest better postoperative outcomes. In posterior decompression surgery, we should pay attention to the correction of SCA, a manageable size.

## Data Availability

The datasets generated and analysed during the current study are availabled from the corresponding author on reasonable request.
